# Finding the optimal balance: father-athlete challenges facing elite Nordic skiers

**DOI:** 10.3389/fspor.2024.1427211

**Published:** 2024-07-18

**Authors:** Max Bergström, Guro Strøm Solli, Øyvind Sandbakk, Kerry McGawley, Stig Arve Sæther

**Affiliations:** ^1^ Department of Sociology and Political Science, Norwegian University of Science and Technology, Trondheim, Norway; ^2^ Department of Sports Sciences and Physical Education, Nord University, Bodø, Norway; ^3^ Department of Neuromedicine and Movement Science, Center for Elite Sports Research, Norwegian University of Science and Technology, Trondheim, Norway; ^4^ School of Sport Science, UiT The Artic University of Norway, Tromsø, Norway; ^5^ Department of Health Sciences, Swedish Winter Sports Research Centre, Mid Sweden University, Östersund, Sweden

**Keywords:** performance, career transitions, dual role, work-life balance, parenthood

## Abstract

**Background:**

In the last decade, a growing body of research has focused on the many aspects and challenges of combining parenthood with elite sport. Although the number of father-athletes is significantly higher than the number of mother-athletes, few studies to date have focused on male athletes’ experiences in a parenting context.

**Aim:**

The aims of the present study were to explore how father-athlete challenges manifest among elite Nordic skiers in Norway, and to better understand how male athletes balance their priorities as they initiate, maintain, and/or discontinue their athletic career as a father-athlete.

**Methods:**

Qualitative data were collected through semi-structured interviews with 10 world-class male Nordic skiers in Norway (3 athletes without a child, 4 current father-athletes and 3 former father-athletes) and the content was analyzed using thematic analysis.

**Results:**

Four main stages were identified in the father-athlete transition: (a) *Expecting incompatibility* (b) *Taking the step,* (c), *The first blow,* and (d) *Finding the optimal balance.* Through these stages the informants expected/had experienced challenges such as performance decline, disturbed sleeping patterns, fear of sickness and role conflicts. To manage these challenges, the father-athletes had developed various strategies to balance their dual roles (e.g., adapting training and competition seasons). Among the benefits, the father-athletes mentioned that they had become more structured, time efficient and ruthless with their priorities, enhanced motivation to train and a better work-life balance.

**Conclusion:**

This study offers valuable insights into father-athlete challenges that can be used to support career longevity and work-life balance among male athletes.

## 1 Introduction

In the last decade, a growing body of research has focused on the many aspects and challenges of combining parenthood with elite sport (1–4). Previous research shows that many female athletes from various sports can return to the same or an even better level of performance after having children (5–8). The number of father-athletes have been reported to be significantly higher than the number of mother-athletes (9), which can be explained by the fact that female athletes are more likely to withdraw from elite sport after childbirth (3, 10). Nevertheless, only a few studies [e.g., (11–14), have focused on the experiences of professional father-athletes. Hence, our knowledge about factors that enable professional father-athletes’ prolongation in sport, as well as perceived challenges and/or benefits when combining parenthood with elite sport, is still strikingly limited.

In a previous study (5) we identified four main challenges specific to mother-athletes: (a) *Biological clock* vs. *peak performance*, (b) *Maintaining fitness* vs. *training safely*, (c) *Receiving support* vs. *facing deselection*, and (d) *Balancing competing mother-athlete demands*. Due to the window of opportunity for building a family, many of the female athletes in that study perceived an inner conflict to prioritize either motherhood or athletic excellence. Other challenges expressed by the female athletes included how to adapt training during pregnancy and balancing the dual mother/athlete roles after childbirth. Additionally, the female athletes felt uncertain about whether they would be able to keep their support from their teams or federations during pregnancy and postpartum. In general, there were expectations of incompatibility surrounding the mother-athlete role. In light of these findings, a natural continuation of our previous work is to explore father-athlete experiences in a similar social context (i.e., Scandinavian Nordic skiers).

Previous research indicates that fatherhood can both improve and impede male athletes’ careers (11, 12, 14). For example, similar to experiences reported by mother-athletes (2, 15), Smith et al. (12) reported that father-athletes welcomed the new responsibilities that came with involved fatherhood, since it helped them to “escape” from their athletic bubble (p.9). Further, fatherhood was perceived to enhance the athletes’ training motivation and time management skills (12). However, the same study also highlighted impediments to performance, such as disruption of sleeping patterns and feeling forced to adapt training routines (e.g., giving up old habits) based on the needs of the child/children. Baetens et al. (14) reported significantly reduced performance among professional male road cyclists after the birth of a child. Other studies have reported that fathers experience guilt when prioritizing sport over family commitments (13, 16). Therefore, the father-athlete role has been described as a trade-off between athletic performance and fatherhood responsibilities (12, 13, 16).

Cross-country skiing is the national sport in Norway and is, therefore, highly popular (17). To compete at an international level, athletes must undertake high volumes of annual training (e.g., 750–950 h) over many years (18, 19), distributed over 11–14 sessions per week (20–22). The sport of Nordic combined is physically and technically demanding, combining cross-country skiing (skating style) and ski jumping on the same day (23–25). Additionally, being a world-class Nordic skier (e.g., cross-country skiing or Nordic combined) requires a large number of travel days in a year (20, 22). The aims of the present study were to explore how father-athlete challenges manifest among elite Nordic skiers in Norway, and to better understand how male athletes balance their priorities as they initiate, maintain, and/or discontinue their athletic career as a father-athlete.

## 2 Theoretical framework

Scandinavian societies have a long tradition of emphasizing social and gender equality, including high political representation of women, public care services for children and elderly, and providing generous parental leave opportunities for both women and men (26). To date, the idea of the “involved and present father”, rather than the father just being the “breadwinner”, as well as shared responsibilities between spouses in the household, are established sociocultural ideals in the Scandinavian culture (27). In the present study, we base our understanding of fatherhood on Fletcher's (16) recent work*.* Similar to Andreasson et al. (27), Fletcher (16) describes a shift in the father role, where today's fathers are faced with new expectations about fathering practices. Here, the “detached” and “breadwinning” father role of the past has been replaced by involved, intimate, caring, and domesticated fatherhood ideals (p.44). The new father role is culturally embedded through social, legal, and moral rights and policies, and has created a new benchmark for fathers to live up to (16). For example, the neoliberal views on parenthood that have emerged in the last decades suggest that parents are solely responsible for the child's upbringing, and that investing in the children's development is the morally right thing to do. This may add pressure to both men and women as they try to live up to perceived expectations in the work, family, and leisure domains (16). Fletcher (16) highlights, that for professional athletes, “for whom their job has a deleterious impact on the amount of time they spend with their family”, absence from the family commitments may be difficult to avoid (p.47). Hence, the potential incompatibility of involved fatherhood and the demands of elite sport (e.g., training, recovery and travel) (15), may create role tensions where professional father-athletes are forced to make difficult decisions about their priorities (12, 13, 16, 27).

## 3 Methods

### 3.1 Participants

To identify suitable informants, purposive sampling was used to recruit three categories of world-class male cross-country skiers or Nordic combined athletes: (a) pre-fatherhood athletes with a wish to have children in the future, (b) current father-athletes, and (c) former father-athletes who had since ended their athletic careers. In line with McKay et al. (28), “world class” was defined as being in the top 20 in world rankings, or top 10 at an Olympics or World Championships. After drafting a list of prospective athletes living in Norway permanently, and with characteristics matching the inclusion criteria, 12 athletes were invited to participate in the study via private telephone or email. Before the informants provided their consent to participate, they confirmed that they matched one of the three categories for inclusion (i.e., having a wish to have or already having had at least one child). In total, 10 athletes (9 Norwegians and 1 international) accepted and fulfilled the study requirements (Table 1). All informants were in a mixed-sex relationship. The international informant had lived in Norway permanently for more than 10 years, spoke Norwegian fluently and had a Norwegian partner.

**Table 1:** Interview guide used in the study.

Interview guide
Athletic background
– Age, sport results, ambitions, team/clubs
– Can you tell me about your plans for the years to come?
Life situation
– What does your life look like?
– Can you describe a typical week?
– Are you able to life from your athletic career?
Father-athlete role
– In what way did/do you expect having a baby affect/will affect your athletic career?
– Can you describe what your expectations are/where before you became a father?
– What would/do/did you find difficult with being/becoming a father-athlete?
– What made you take the step?
– What would/do/did you need to make the father-athlete role work?
– In what way has/did the father-athlete role affected your athletic career?
– How did being a father affect your training routines/competition program?
– What kind of father/partner would you like to?
Significant others
– What reactions would/do/did you get from your team/coach/sponsors when you told them that you were going to become a father?
– What do you think your family and friends (would) think/thought about you continuing your athletic career as a father?
– What kind of support would/do/did you need?

### 3.2 Data collection and analysis

Data were collected through semi-structured interviews lasting 19–41 min (M = 26.3; SD = 5.9). Since the informants were based in various locations in Norway or abroad (e.g., on training camps), the interviews were conducted using online video conference software (Zoom Video Communications, Inc.). Compared to physical interviews, collecting data remotely is more time efficient and flexible for the researchers and the informants (29). To facilitate the data collection, the interview guide included topics relating to the informant's athletic background, family life, economic situation, and preconceptions, experiences, and perceived challenges relating to the father-athlete role. Some of the questions from the interview guide were: (a) In what way did/do you expect having a baby affect/will affect your athletic career? (b) What do/did you find difficult with being/becoming a father-athlete? (c) What would/do/did you need to make the father-athlete role work? The international informant was given the opportunity to conduct the interview in English but since he spoke Norwegian fluently, he insisted on completing the interview in Norwegian. Hence, all interviews were conducted in Norwegian.

All interviews were recorded and transcribed and the content was analysed according to the six steps of thematic analysis (30, 31): (1) familiarizing yourself with the data, (2) generating initial codes, (3) searching for themes, (4) reviewing themes, (5) defining and naming themes, and (6) producing the report. After transcribing the interviews, the first and second authors read the transcripts to get a general sense of the material (step 1). Next, interesting features were bunched roughly into different categories (step 2). These preliminary themes were labelled in step 3. In step 4, the first and second author reviewed the preliminary themes after discussing them from different research perspectives and implications. In step 5, the themes were refined and labelled into four main themes: (a) *Expecting incompatibility* (b) *Taking the step,* (c) *The first blow,* and (d) *Finding the optimal balance.* In the final step, step 6, corresponding quotes that reflected the themes and the study aim were selected and presented in the report. To ensure peer validity, all authors discussed various perspectives and interpretations of the themes. Additionally, to avoid any potential misunderstandings when translating selected quotes from Norwegian to English, the co-author with English as a first language (K.M.) agreed on the translations of the selected quotes.

### 3.3 Ethical statement

The prospective informants were presented with the study objectives in a letter with all necessary information (e.g., interview topics, procedures, and confidentiality) before they provided their informed consent to participate. All informants were informed that participation was voluntary and that they could withdraw at any time during the process until the findings were published. After the interviews, each informant was given an opportunity to add, remove, or correct the data from their interview transcript. All informants have been assigned pseudonyms to minimize the potential risk for being identified from the selected citations. Additionally, specific information that could reveal the identity of the informant, such as cities, names of children, partners, teammates or coaches, or details about athletic achievements, was replaced or removed. The study was carried out according to the Declaration of Helsinki and approved by the Norwegian Social Sciences Data Services (ref. 995878).

### 3.4 Rigour

In line with guidelines for high-quality qualitative research advocated by Tracy (32), we ensured that the eight criteria: worthy topic, rich rigor, sincerity, credibility, resonance, significant contribution, ethics, and meaningful coherence were adhered to. The authors’ backgrounds provide unique expertise, influencing the qualitative interpretation of these data, and our combined experience as athletes, coaches and researchers spans over many years. Moreover, some of us have been top-level athletes in endurance sports and others have closely collaborated with world-class endurance coaches and athletes, many who faced the challenges investigated in this study, both within the Olympic Training Center (Olympiatoppen) and various national sports federations in Norway and Sweden. This demonstrates our in-depth understanding of the subject, and we contend that this wealth of experience and insider perspective uniquely qualifies us to collect and interpret the data for this study. Additionally, the authors’ networks facilitated the process of identifying and contacting potential informants. For example, the authors’ backgrounds enabled them to contact athletes directly (e.g., personal connections) or through their team staff or sport federations.

## 4 Results

**Figure 1:**
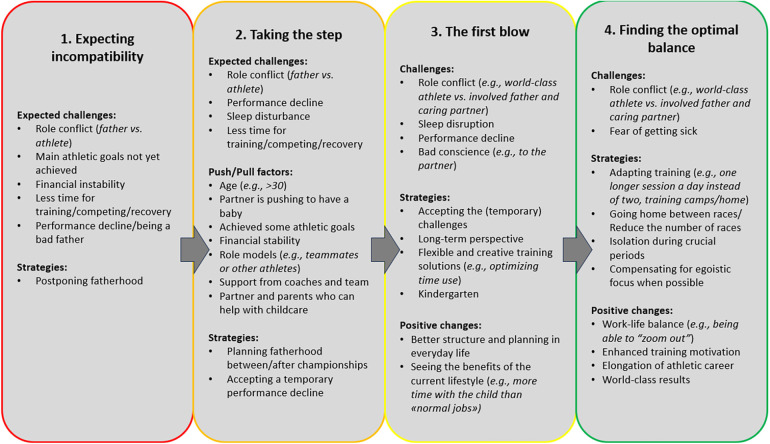
A summary of the four main father-athlete challenges faced by the male athletes.

Four main themes reflecting specific father-athlete challenges were identified (Figure 1). The first theme, *Expecting incompatibility,* reflects the athletes’ thought processes prior to fatherhood and how their priorities were negotiated. The second theme, *Taking the step,* reflects athletes’ motives for initiating the father-athlete role. The third theme, *The first blow,* highlights the challenges perceived by father-athletes during their first year as a parent. The fourth theme, *Finding the optimal balance,* relates to how the current and former father-athletes combine/d their dual roles over time, as well as the former father-athletes’ motives for terminating their athletic careers.

### 4.1 Expecting incompatibility

All except one informant (Gustav) described ambivalent feelings about the process of becoming a father-athlete. For example, one of the pre-fatherhood athletes (Oliver) expressed a wish to have children at some point, but felt unsure about how being a father would impact his athletic career:

I can’t really see that it would be ideal [*for my athletic career*], to be frank. I would find it difficult with elite sport and training twice a day and with the total load of having children. I have quite a few nephews and nieces and so on…I see how much is actually required, so yes…I simply cannot see that it would be an advantage [*for my athletic career*]. […] when you have your best years [*as an athlete*] ahead of you, I find it difficult to prioritize children.

One of Oliver's main concerns was that he would have to give up his own “privileges” (e.g., training and recovery time), since he also felt that he would want to assist his partner with the childcare. Further, with over 100 travel days per year, he felt that he would have to reduce his participation in World Cup races and training camps to be the partner and father that he wanted to be. As mentioned in the next quote, Oliver believed that he still had his best years as an athlete ahead of him. Being in this stage of his athletic career made him less willing to “gamble” with his future. However, even though Oliver felt somewhat negative towards the prospect of becoming a father at the time of the interview, he had started to negotiate with himself (e.g., he expressed a willingness to try towards the final years of his athletic career) and had started a process of preparing himself mentally for a future as a father-athlete:

The thought [*of starting a family*]*,* it comes almost every day and the older you get, the more you try to become more structured, more disciplined, because you know that you have to try to make the best of the privilege and the free time you have as a young person. […] I toyed a bit with the idea from a long-term perspective…I have toyed with the idea that I will actually have children while I’m still an athlete, but maybe it will be a way of tapering off, that I am willing to try it as long as it goes well.

Matheo, another of the pre-fatherhood athletes, believed that becoming a father could be a positive life event that might enhance his athletic career by providing positive energy:

I guess that if I’m going to think about having children then it's a great joy […]. It's a bit like falling in love […] things become much easier in the mind, mentally. That's probably the thing about having children, and that mentally it becomes quite a lot more positive than how “normal” everyday life had turned out to be [*otherwise*].

However, even if his partner was ready to have a baby and was willing to take more responsibility for the child so that he could continue his athletic career, he felt unsure how it would affect his athletic performance, but also whether he, similar to Oliver, could be the father he wanted to be:

When I have children, I feel that I don't want to deprioritize them and be left with a bad feeling that it is me who is the main focus all the time and that they are not looked after and don't get the upbringing that they deserve. Now it is one and a half years until the World Championships, so I want to spend all my time and energy on me and […] be the best I can be for that […]. In my head, I believe that *[having children]* isn't possible if I am going to perform at my best.

Matheo was clearly struggling to come to terms with how he would combine the dual roles of being a world-class athlete and an involved father. With a home World Championships coming up in 18 months, which he described as his main career goal, he deemed having children before this as simply too risky. Further, in contrast to Oliver, Matheo expressed that his income from skiing was not enough to provide for a baby. Both Matheo and Oliver had recent experiences of teammates who had become fathers and who (in their opinions) did not perform at the same level after this. While Matheo did not believe his coach would be against him becoming a father, from a performance perspective he did not feel his coach would think of it as beneficial for his athletic development. In Oliver's case, other pre-conditions were absent. He believed that his coach would indeed support him and that he had financial stability, but his partner had a fulltime job and could not take care of a baby in the near future. Additionally, both Matheo and Oliver expressed that they still felt relatively young (both were less than 30 years old) and therefore did not perceive any time pressure to have children.

### 4.2 Taking the step

The current and former father-athletes had experienced similar doubts as Matheo and Oliver. Sander (a former father-athlete), who had been doubtful for several years before he “dared” to become a father-athlete, mentioned his age and career achievements as crucial turning points:

Both my partner and I had reached 30. The time was kind of right […]. My partner might have wanted to have children a bit sooner, but then we ended up buying a dog instead *(laughter)* just to delay it […]. It was actually a bit up to me when we wanted to have children because we have seen athletes in the past who have had children and it has not always been positive, so I was a bit anxious about that transition. Yes, I was a bit anxious, I was. Then I won that gold medal and by then I had already achieved a lot of what I had dreamed of, so then we dared to take that step.

One of Sander's biggest concerns had been the increased risk of catching an infection (e.g., from kindergarten), which could easily jeopardize his entire competition season. His concerns were shared by Henrik (a current father-athlete):

I understood that having a child will have an impact, in the sense that there will be less rest, call it an athletic risNk because you don’t know how it will be with sleep […]. I have always wanted to be a father […]. I had reached 30 years old, was in a stable relationship, I was financially stable, I had achieved a lot in sport and so it felt like a natural step.

Henrik's age and previous athletic achievements, in combination with several other factors (e.g., financial stability and being in a stable relationship), had been determining factors for him to take the step to become a father-athlete. In contrast to Matheo and Oliver, both Henrik and Sander had witnessed some of their teammates manage the father-athlete transition successfully. Moreover, they mentioned having their parents and/or partners’ parents living nearby as factors that had made the transition smoother, since it provided them with extra support (e.g., babysitting). For Olav (a current father-athlete), this was considered crucial for him to be able to continue his athletic career as a parent. For example, he and his partner moved to another town to be closer to his partner's parents, even though this had also meant leaving his training environment (e.g., training facilities, teammates, and coaches). Although he had found it difficult to leave, he believed that the decision would prolong his athletic career. For Alfred (a current father-athlete), his attitude towards becoming a father-athlete had mainly changed due to an ultimatum from his partner:

Initially I wasn’t going to have children while I was an athlete, but then I met a woman who really wanted children and I really wanted to be with her. Then it was like…she was quite clear that she wanted children. I tried to put it off for as long as I could, but there came a point where I couldn’t any longer and then it was either that it will end or we would have children, so then it was a simple choice.

Hence, the informants experienced different turning points, which changed their initial skepticism for initiating the father-athlete role.

### 4.3 The first blow

The current and former father-athletes’ partners had played a key role in enabling them to continue as world-class athletes by taking the main responsibility for the childcare. However, even if this facilitated the elongation of their athletic careers, all informants had found themselves in a difficult position where they experienced a role conflict between being a world-class athlete and an involved father and caring partner. On one hand, they were aware of the demands of their sport (e.g., high training volumes, significant rest time, an egotistic focus and time away from home), while on the other hand they wanted to support their partners with the childcare duties (e.g., getting up in the night if the baby woke or taking care of ill children). Henrik, a current father-athlete, and first-time father, had experienced the first year as challenging in multiple ways:

It has been challenging because I’ve performed at the level I have and have the goals I have and the experience I have, so I know incredibly well what it takes in terms of sleep and recovery and when you realize yourself that you don’t get it, you understand in a way that this isn’t good enough […]. I would probably have performed better this last winter if we hadn’t had children […] I should be honest about that […] I performed well, but I did not perform as well as I did the year before and as I think I will do this year.

Having a baby had resulted in a decline in his performance, which he felt dissatisfied about. At the same time, he had a bad conscience towards his partner. Henrik explained that even if his partner often reminded him not to contribute too much in the household, and that he should focus on his athletic goals (with a home World Championships 18 months away), he still wanted to “take some weight off her shoulders”:

It’s [*the father-athlete role conflict*] very difficult to do something about and you don’t want to do anything about it either, because that means putting even more of the burden on your partner and it's also out of the question, so you kind of get caught there, that you feel you aren't really good enough anywhere because you would have liked to have contributed more, but at the same time you know that you are contributing too much in relation to performing [*in sport*] […]. It's a bit of a strange intermediate position. It's a bit like being between a rock and a hard place…

Henrik described that he had learned to be more accepting of the fact that he was unable to follow his training plan in detail during the first year of fatherhood, and that he had to be more flexible and creative with his training sessions (e.g., going for a jog with the stroller so his partner could sleep). Further, he described that being a parent had made him prioritize the most important parts of his training plan. However, now that his child had recently started kindergarten and slept through the night, he felt more optimistic about the upcoming competition season (as mentioned in his first quote).

Apart from having a supportive partner, kindergarten was highlighted as beneficial for all the informants with children. After their partners returned to work following maternity leave, the father-athletes described that they could take responsibility for taking their children to kindergarten before their training sessions. Kindergarten also enabled the father-athletes to get some rest in the middle of the day, which meant they could play with their children in the evenings. Over time, Henrik also saw some benefits of his lifestyle compared to fathers with “normal jobs”, such as being able to spend more time with his family during the periods at home. Nevertheless, because of the challenges he had experienced during the first year, Henrik was clear that he and his partner would not have any more children before he had ended his athletic career.

Ulrik, also a current father-athlete and first-time father, described that becoming a father had been a highlight of his life. Like Henrik, Ulrik experienced a decline in performance during his first year as a parent. However, he felt confident that this wouldn't affect his career negatively in the long term. Further, he and his partner had planned to have their baby between two Winter Olympic Games to minimize any potential negative impacts on his athletic career. Ulrik also described that having a child had made him more structured in his daily training, which he found positive:

A year ago I got up when I wanted and trained when I wanted. Now there are slightly more fixed times to train before 8.30 in the morning and be finished by 11.30–12.00 at the latest, so that I can relieve my partner between training sessions. […] In the long run I didn’t think that it [*having children*] would really affect my career that much. It requires more structure, more structure in everyday life and a bit more planning longer in advance if there is a period where I will be away, periods of travel, competitions, and training camps […].

As Ulrik mentions, taking weight off his partner's shoulders was also important to him. Notably, all the current and former father-athletes perceived a similar role conflict (i.e., being a world-class athlete and an involved father and caring partner). This concern was also expressed by Oliver and Matheo (pre-fatherhood athletes) as one of their main reasons for postponing fatherhood.

### 4.4 Finding the optimal balance

Although the father-athletes’ athletic careers often tended to be prioritized, the informants developed ways to compensate for the time they invested in fulfilling their own athletic ambitions. For example, for the upcoming competition period Ulrik had decided to stay at home during some of the World Cup weekends to spend time with his family. Three of the informants had started training one longer session every day instead of two separate sessions, to make their training more accommodating of the family logistics. Magnus, a former father-athlete for 7 years, only trained five days a week to be able to spend the weekends together with his children. He had also tried to come home between races in the competition season when it was possible. Because of his fear of getting sick, Sander (a former father-athlete) avoided going to the kindergarten during the specific preparation period and competition season, but he compensated for this by contributing more during the spring and the summer months. Conversely, Olav (a current father-athlete) and Gustav (a former father-athlete) tried to compensate continuously during the whole year (e.g., between training camps) by taking the children to kindergarten, preparing meals and putting the children to bed at night. Unlike Magnus, who found that doing most of his training in his hometown had been a key element for his continuation as a father-athlete (e.g., to optimize time and minimize time away from his family), Gustav, who had been a father-athlete for 6 years, described that going away regularly on training camps had been his formula for success by enhancing motivation:

After a while you get a bit tired of going to training camps and being in the same places and staying in a hotel room, but suddenly it was a “holiday” *(laughter)* […]. When I became a father, I actually appreciated staying in a hotel even more, having meals prepared every day, not having to do any dishes…you get the idea…I think it's very positive to have that change. For me, it was extremely important to be able to go to the training camps and have full focus both on training, but also on recovery. To enjoy staying in a hotel and eating breakfast and not having any children, diapers or dishes. It was almost as if I felt that “now I can continue even longer” [*with my athletic career*] because I had a completely different approach to going to training camps and races […] at the same time I was very much looking forward to coming home to my children. It put things into perspective and created a nice balance between being an elite athlete and egoist and being a family man.

Gustav had not only managed to find a work-life balance by being a world-class athlete and an involved father and caring partner, but he had also found a way to combine these roles successfully. For example, he describes a feeling of renewed motivation about his athletic career and more energy for his family life. Additionally, having a family had enabled him to zoom out from the elite sport bubble, which had helped him to cope with bad results or other disappointments in sport. His solution had not been possible without the support of his wife, who he described had been a “single mom” for half of the year. Just like the other informants, Gustav had a bad conscience about prioritizing his own needs. Yet, he felt that he had been able to compensate during the periods that he was home. While Magnus and Gustav achieved some of their best results (e.g., winning international medals) in their careers as father-athletes, other informants like Sander struggled:

In an ideal world, I would have been able to train and be with my family at the same time. It's not quite optimal. It's a bit like that, if you have first chosen to be an elite athlete and have ambitions to be the best at what you do, then you must set some priorities. Not everything is fun, but that's just the way it is. […]. This year I celebrated Christmas alone at the cabin because they [*the children*] were at home ill. You have to constantly make adjustments. If someone at home is sick, then I’ve mostly gone away.

To Sander, combining the demands of sport (e.g., high training volumes, travel and avoiding infections) and the needs of the family (e.g., taking care of his children when they were ill) was perceived as an unsolvable challenge. No matter what he chose to prioritize (e.g., his family or athletic career), he felt that he was doing something wrong since the other life domain was affected negatively. After 2.5 years as a father-athlete and shortly after having his second child, Sander chose to retire from sport:

If the children become ill then in a way it feels a bit wrong to go and stay in the cabin for two weeks. Then she *[my partner]* is home with two sick children, but then it would [*also*] have been wrong to stay at home if they were ill. It's not right no matter what you do […] it's like doing it by half. Half at home and half an athlete. […] You won’t win any World Championship golds and you won’t take any medals at the World Championships if you only do it by half and elite sport is fun if you’re good, but not so much fun if you don’t perform well […]. I didn’t want to continue [*my athletic career*] and be a mediocre skier.

Hence, balancing the father-athlete experience seemed to vary between individuals, depending on their expectations of their athletic and family life domains. For Sander, neither being a mediocre skier nor feeling guilt towards his family were acceptable alternatives.

## 5 Discussion

### 5.1 Becoming a father-athlete

Similar to previous research conducted on both male and female athletes [e.g., (2, 5, 12)], our findings show that combining elite sport with parenthood can be challenging (e.g., creating role conflicts and a decline in performance) but also rewarding (e.g., by enhancing motivation, improving work-life balance, and prolonging the athletic career). In the process of initiating the father-athlete role, all except one informant (Gustav) described ambivalent feelings towards combining fatherhood with elite sport. Negative factors such as a decline in athletic performance, sleep disruption, reduced training time, increased risk of infection and role conflict (i.e., being a world-class athlete and an involved father and caring partner) were reported/expected by the informants. Even though previous research indicates that female athletes are more likely to have multiple identities than their male peers (11, 33), our study shows that the informants strived to be involved fathers and caring partners, and fulfill their athletic ambitions. Similar findings have been reported in other studies of recreational male athletes (13, 16, 34). The perceived role tensions seemed to arise amidst a clash between the informants’ beliefs about the sport-specific demands and fatherhood. For example, the informants’ understanding of fatherhood aligns with previous research on fatherhood in Scandinavia [e.g., (27)], whereby being involved in the child's life and sharing duties with the partner in the households are established ideals. Their expectations also align with Fletcher's (16) description of involved, intimate, caring, and domesticated fatherhood ideals. Further, the informants expressed sport-specific demands (e.g., training volume, recovery time and traveling) similar to those reported by other world-class cross-country skiers [e.g., (20–22)]. This could be one explanation for the pre-fatherhood athletes’ preconceptions of role incompatibility. For example, six informants expressed that they had or were trying to postpone parenthood for as long as possible, to avoid negative effects on their athletic careers (e.g., upcoming championships). A recent study of 299 professional male road cyclists showed that cycling performance was significantly reduced after the birth of a child (14). This indicates that the concerns expressed by the informants in the present study were legitimate. Consistent with Bergström et al.'s (5) study on female athletes, coming to terms with the risk of a potential decline in performance seemed to be easier for the informants who had already achieved some of their main athletic goals (e.g., winning international medals). In contrast, two of the pre-fatherhood informants (Oliver and Matheo), who felt that they had their best years as athletes ahead of them, were less willing to take unnecessary risks. Additionally, the informants felt that several other factors had to be in place (e.g., financial stability, the timing to championships and partner/parental support) to initiate the father-athlete role. This finding aligns with previous research showing that many individuals consider moral aspects (e.g., being able to provide the best start for their child) as they negotiate the right time to start a family (16, 34, 35). However, our findings show that such factors were weighted differently by each informant. For Al fred, his partner's ultimatum had been the main reason for initiating the father-athlete role.

Unlike the mother-athletes in our previous study (5), who expressed concerns about losing support from their team or sponsors when initiating the mother-athlete transition, the informants in the present study did not report such concerns. On the contrary, two informants mentioned that they had or would have benefits from becoming fathers due to the positive image presented to sponsors. This aligns with findings by McGannon et al. (11), who explored media representation of a father-athlete in professional tennis and identified images of the “good father” and the “new and improved athlete father”. Further, none of the male informants expressed any concerns of risking deselection if they would become fathers, and they believed that their coach and/or team management would support them. By comparison, Bergström et al. (5) reported that female athletes experience uncertainty about maternity provisions from their professional teams, sport federations and sponsors. Unlike female athletes, who typically have fewer mother-athlete peers and role models, all informants in the present study had at least one teammate who had made the parenthood transition before them and the current and former father-athletes expressed that seeing other teammates making the father-athlete transition successfully had encouraged them. However, two of the pre-fatherhood athletes had witnessed teammates struggling to combine the dual roles. For these informants, their teammates acted as conformation that it would be wiser to postpone fatherhood until they had achieved at least some of their athletic goals. Hence, the experiences of other athletes, whether positive or negative, seemed to influence the athletes’ thought processes.

### 5.2 Maintaining the father-athlete role

One of the main findings of the present study was that the father-athletes, similar to mother-athletes, struggle to balance their dual roles and experience feelings of bad conscience by not focusing enough on parenthood and/or sport. This aligns with Cohen (13), who reported that male recreational Ironman triathletes (“Iron Dads”) attempting to combine sport as a hobby with father, husband and full-time employee roles often suffer from sporting guilt. Similar to the Iron Dads (13), the male informants in the present study expressed that they had to juggle several identities (e.g., worldclass athlete, father and husband), and that improving their performance in sport was perceived to diminish their level of involvement as a parent. This finding also aligns with experiences reported by other female [e.g., (5, 16, 31)] and male athletes [e.g., (12, 16)]. Consistent with both Smith et al. (12) and Cohen (13), the informants in our study described a trade-off between their athletic and family commitments. For example, Cohen (13) described how the Iron Dads developed mechanisms for balancing their absence from family life (e.g., by adapting training times, combining family holidays with triathlon races and competing in shorter distance races). Similar “give-and-take” practices (12) could be identified among the current and former father-athletes in the present study, who perceived that fatherhood could both improve and impede their athletic performance. For example, reduced time for training, sleep disruption and fear of getting sick were perceived as particularly challenging during the child's early years. Therefore, one informant (Gustav) described travelling without the family to training camps or competitions as a relief, as well as a good way for him to find a better work-life balance that enhanced his motivation in both life domains. Similar give-and-take negotiations were observed among the pre-fatherhood athletes. For example, Oliver reflected on potentially reducing time away to be the future father he wanted to be. These findings indicate that the informants had internalized similar fatherhood ideals and values as those described by Fletcher (16) (e.g., involved, intimate, caring, and domesticated fatherhood ideals) and tried to live up to this benchmark.

Variation between the informants’ give-and-take practices indicates that every individual must find arrangements that work with the needs of their family and their athletic commitments. As stated by Henriksen et al. (36) in relation to dual careers, “finding an optimal balance is seen as an ongoing process that never truly ends because the circumstances evolve” (p.9). The mother-athletes interviewed by Bergström et al. (5) felt that the compatibility between life commitments was improved once their children started kindergarten, at approximately 18 months old. Similar findings were reported by the father-athletes and former father-athletes in the present study. Further, our findings align with Smith et al. (12), who described “the trade-off between fatherhood and athletic performance as a dynamic process that is dependent on a multitude of factors that contribute to an ebb and flow of priority and time management between fathering and athletic commitments, including child(ren)'s age(s), future goals and/or previously accomplished career milestones, and time of year in relation to competitive season” (p.15). Our findings show that the fathers who maintained dual roles for the longest duration (Gustav and Magnus) had found ways to cope with and balance various father-athlete challenges that he had encountered. Our study also showed that role incompatibility was perceived as one of the main concerns among the pre-fatherhood athletes and, for one of the former father-athletes (Sander), was described as the main reason for discontinuing his athletic career. These findings align with Fletcher (16), who outlined that fathers who feel unable to combine the roles (e.g., work, sport and family) may discontinue their participation in sport. Consistent with our findings, Fletcher (16) also showed that those fathers who felt supported by their partner or other family members perceived their involvement in sport to be much easier. Hence, depending on what challenges athletes perceive, and whether these can be managed by the athlete and other stakeholders (e.g., partner, other family members, team, sport federation, sponsors, kindergarten), the father-athlete role can prolong the athletic career, promote the work-life balance and enhance training motivation needed to achieve athletic goals. However, if the athlete is unable to cope with the challenges, the result may be the opposite (e.g., role tensions, performance decline, lower motivation and discontinuation from sport).

## 6 Conclusions

In this study we explored how father-athlete challenges manifest among elite Nordic skiers in Norway, with a view to better understand how male athletes balance their priorities as they initiate, maintain, and/or discontinue their athletic career as a father-athlete. We identified four main stages in the father-athlete transition: (a) *Expecting incompatibility,* (b) *Taking the step,* (c) *The first blow,* and (d) *Finding the optimal balance.* In these stages, the informants expected or had experienced challenges such as role conflicts (e.g., father vs. athlete), performance decline, disturbed sleeping patterns and fear of sickness. To manage these challenges, the current and former father-athletes developed various strategies to balance their dual roles (e.g., adapting training and competition seasons). Among the benefits, the informants perceived that fatherhood had made them more structured, time efficient and ruthless with their priorities. Other perceived athletic improvements related to fatherhood included enhanced motivation to train and a better work-life balance. The prolongation of the informants’ athletic careers was enabled by support from their partner or other family members. This study offers valuable insights into father-athlete challenges that can be used to support male athletes who wish to successfully prolong their athletic careers whilst also having children. Given our relatively small sample size, future studies could consider using quantitative approaches to further our understanding of father- and mother-athlete challenges and opportunities. Furthermore, future studies should explore the father-athlete topic within other social contexts (e.g., other sports and cultures).

## Data Availability

The raw data supporting the conclusions of this article will be made available by the authors, without undue reservation. Requests to access the datasets should be directed to max.v.j.bergstrom@ntnu.no.
