# Supplementary material for: Finding the optimal balance: father-athlete challenges facing elite Nordic skiers
Source: Front Sports Act Living. 2024 Jul 18;6:1427211. doi: 10.3389/fspor.2024.1427211 (PMC11291440; doi:10.3389/fspor.2024.1427211)
Supplement: Supplementary file 1 [file Presentation1.pptx]

## Slide 1
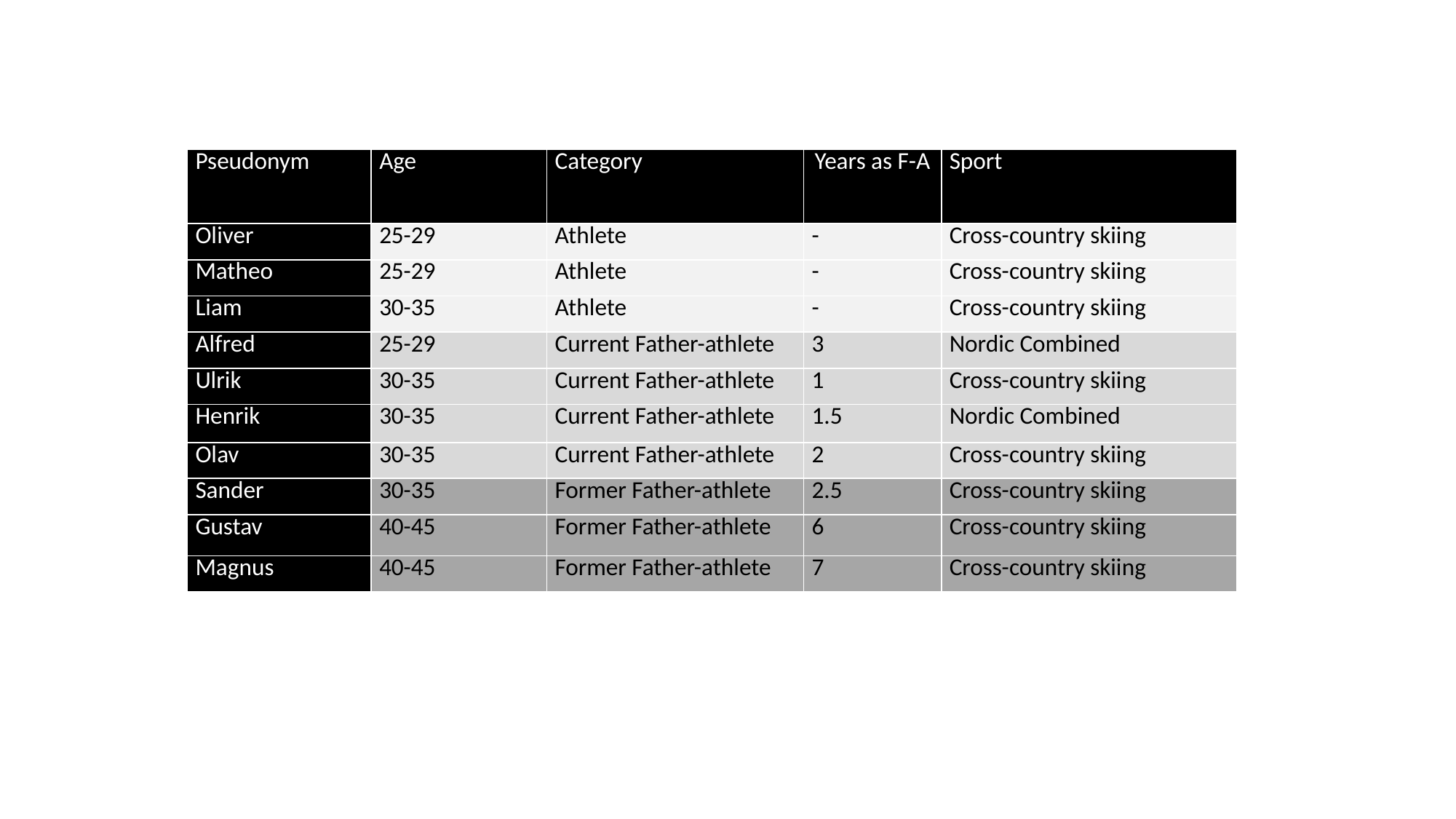

| Pseudonym | Age | Category | Years as F-A | Sport |
| --- | --- | --- | --- | --- |
| Oliver | 25-29 | Athlete | - | Cross-country skiing |
| Matheo | 25-29 | Athlete | - | Cross-country skiing |
| Liam | 30-35 | Athlete | - | Cross-country skiing |
| Alfred | 25-29 | Current Father-athlete | 3 | Nordic Combined |
| Ulrik | 30-35 | Current Father-athlete | 1 | Cross-country skiing |
| Henrik | 30-35 | Current Father-athlete | 1.5 | Nordic Combined |
| Olav | 30-35 | Current Father-athlete | 2 | Cross-country skiing |
| Sander | 30-35 | Former Father-athlete | 2.5 | Cross-country skiing |
| Gustav | 40-45 | Former Father-athlete | 6 | Cross-country skiing |
| Magnus | 40-45 | Former Father-athlete | 7 | Cross-country skiing |
