# Supplementary figures and images for: Finding the optimal balance: father-athlete challenges facing elite Nordic skiers
Source: Front Sports Act Living. 2024 Jul 18;6:1427211. doi: 10.3389/fspor.2024.1427211 (PMC11291440; doi:10.3389/fspor.2024.1427211)

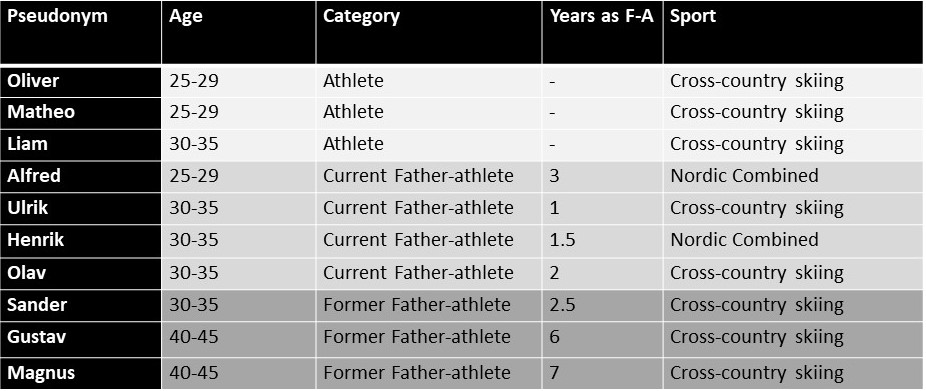

Supplement: Supplementary file 2 [file Image1.jpeg]
